# White Paper by the European Society for Swallowing Disorders: Screening and Non-instrumental Assessment for Dysphagia in Adults

**DOI:** 10.1007/s00455-021-10283-7

**Published:** 2021-03-31

**Authors:** Renée Speyer, Reinie Cordier, Daniele Farneti, Weslania Nascimento, Walmari Pilz, Eric Verin, Margaret Walshe, Virginie Woisard

**Affiliations:** 1 Department Special Needs Education, University of Oslo, Oslo, Norway; 2 School of Occupational Therapy, Social Work and Speech Pathology, Faculty of Health Sciences, Curtin University, Perth, Australia; 3 Department of Otorhinolaryngology and Head and Neck Surgery, Leiden University Medical Centre, Leiden, The Netherlands; 4 Department Social Work, Education and Community Wellbeing, Northumbria University, Newcastle upon Tyne, UK; 5 Audiologic Phoniatric Service, ENT Department, AUSL Romagna, Infermi Hospital, Rimini, Italy; 6 Gastrointestinal Physiology Laboratory, Department of Surgery, Hospital de Mataró, Universitat Autònoma de Barcelona, Mataró, Spain; 7 Department of Otorhinolaryngology, Head and Neck Surgery, Maastricht University Medical Center, Maastricht, The Netherlands; 8 School for Mental Health and Neuroscience, Maastricht University, Maastricht, The Netherlands; 9 Dept of Physical and Rehabilitation Medicine, Rouen University Hospital, Rouen, France; 10 Department of Clinical Speech and Language Studies, Trinity College Dublin, Dublin, Ireland; 11 Voice and Deglutition Unit, Department of Otorhinolaryngology and Head and Neck Surgery, Larrey Hospital, University Hospital of Toulouse, Toulouse, France; 12 Oncorehabilitation Unit, Toulouse Universitary Cancer Institute, Oncopole Hospital, Toulouse, France

**Keywords:** Measures, Diagnostic accuracy, Validity, Reliability, Responsiveness, Psychometrics

## Abstract

This White Paper by the European Society for Swallowing Disorders (ESSD) reports on the current state of screening and non-instrumental assessment for dysphagia in adults. An overview is provided on the measures that are available, and how to select screening tools and assessments. Emphasis is placed on different types of screening, patient-reported measures, assessment of anatomy and physiology of the swallowing act, and clinical swallowing evaluation. Many screening and non-instrumental assessments are available for evaluating dysphagia in adults; however, their use may not be warranted due to poor diagnostic performance or lacking robust psychometric properties. This white paper provides recommendations on how to select best evidence-based screening tools and non-instrumental assessments for use in clinical practice targeting different constructs, target populations and respondents, based on criteria for diagnostic performance, psychometric properties (reliability, validity, and responsiveness), and feasibility. In addition, gaps in research that need to be addressed in future studies are discussed. The following recommendations are made: (1) discontinue the use of non-validated dysphagia screening tools and assessments; (2) implement screening using tools that have optimal diagnostic performance in selected populations that are at risk of dysphagia, such as stroke patients, frail older persons, patients with progressive neurological diseases, persons with cerebral palsy, and patients with head and neck cancer; (3) implement measures that demonstrate robust psychometric properties; and (4) provide quality training in dysphagia screening and assessment to all clinicians involved in the care and management of persons with dysphagia.

## Introduction

The terms *Dysphagia* and swallowing disorders are used interchangeably in the literature; however, achieving international consensus on defining dysphagia is more challenging. Despite there being no universally accepted definition, a commonly used description of dysphagia is the dysfunction of one or more parts of the swallowing apparatus including the mouth, the tongue, the oral cavity, the pharynx, the airway, and the oesophagus and its upper and lower sphincters, often due to anatomical or structural deficiencies or abnormalities [1]. Swallowing is not only essential for nutritional intake, but it is also directly involved in the management of internal secretions from the upper and lower aerodigestive tracts (e.g. saliva, bile, and nasal, tracheal or bronchial secretions). Dysphagia is associated with a broad spectrum of underlying congenital or acquired conditions which is influenced by both age and comorbid diseases [2]. Dysphagia is one of the leading causes of death and morbidity for older persons, children and adults with neurological disorders (e.g. cerebral palsy, stroke, dementia), or head and neck cancer patients, due to serious complications such as malnutrition, dehydration, and aspiration pneumonia [3, 4].

Dysphagia is a symptom or collection of symptoms resulting from underlying impairments and disorders in the highly integrated neuromotor sequence of swallowing, from forming a cortical decision through to the realization of motor action with associated sensory modulation, defined by series of events that involve the digestive and upper respiratory tract and the interaction of various anatomical structures along the pathway [5]. Dysphagia may modify the preparation, propulsion, and/or transit of the bolus through the upper digestive pathways and reflects abnormalities in transit and/or airway protection. A distinction can be made between oropharyngeal and oesophageal dysphagia.

Not having reached consensus on a universal definition of the construct of dysphagia impacts on making an accurate estimation of the *prevalence* of dysphagia, as it influences what criteria need to be met to fall within the parameters of the definition. A systematic review on the prevalence of dysphagia in the general population indicated marked variability in prevalence rates, which ranged between 2.3 and 16% [6]. Another review demonstrated that the prevalence of dysphagia after stroke and in patients with Parkinson’s disease, traumatic brain injury, and community-acquired pneumonia was high (8.1–80%, 11–81%, 27–30%, and 91.7%, respectively) [7]. The high variability in the retrieved prevalence data can be explained by discrepancies regarding dysphagia definitions, underlying diseases being at different stages with variable sequelae and using different measures to determine the presence of dysphagia. The impacts of definitional issues on determining accurate prevalence rates are twofold. First, definitional inconsistencies will have an impact on aspects of validity, reliability, and generalizability of prevalence numbers. Second, screening or assessments of dysphagia operationalizes the underlying construct being measured, and definitional inconsistencies will invariably influence what and how dysphagia is being measured. Despite the implications of definitional challenges, given the high prevalence of dysphagia [6, 7], the serious complications associated with it, and costs to healthcare [8], systematic screening of at-risk populations such as acute stroke patients should be central to healthcare strategies to improve early intervention and clinical outcomes in these clinical populations. Moreover, the high prevalence of dysphagia underscores the need for standardized, reliable, and valid methods for the screening and assessment of dysphagia.

The purpose of screening is to identify persons at risk of dysphagia. Patients who are identified as being at risk through screening are typically referred for further assessment and, where needed, related assessment including oral health and gastroesophageal reflux. Both screening and assessment need to be evidence-based and feasible to administer. Only screening with sufficient diagnostic performance and assessments with robust psychometric properties should be used clinically and in research. For example, using screening tools with poor diagnostic performance may result in missing people at risk for dysphagia (false positives) or lead to unnecessary use of limited resources by assessing those who are not at risk of dysphagia (false negatives). Similarly, implementing assessments with poor psychometric qualities will undermine evidence-based practice and research as current health status or intervention effects cannot be objectified if measures lack robustness in validity, reliability, or responsiveness [9, 10]. Selecting the best evidence-based screening and assessments for dysphagia is an essential step in the management of dysphagia.

The purpose of this white paper by the European Society for Swallowing Disorders (ESSD) is to report on the current state of screening and non-instrumental assessment for dysphagia in adults. The measures that are available, how to select screening tools and assessments, and gaps in research that still need to be addressed in future research will be discussed.

## Screening

### Screening in a Nutshell

**Fig. 1:**
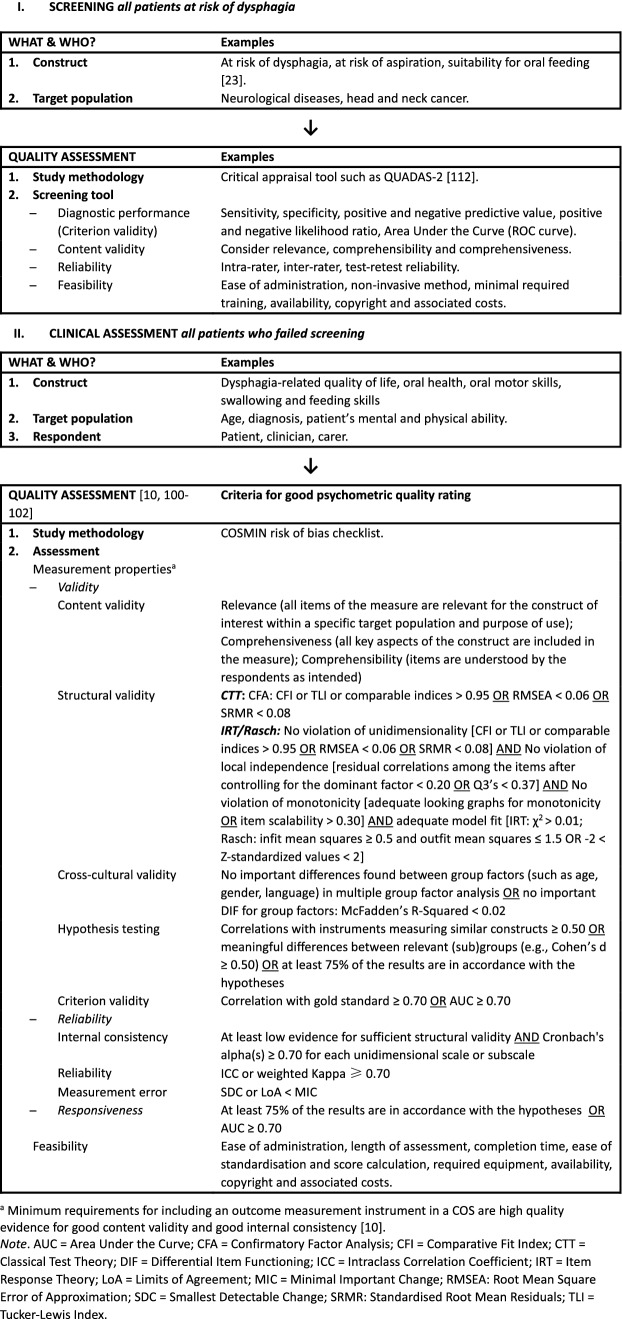
Flow chart for selection of screening tools and clinical assessments

*Screening* is generally accepted as the first step in the management of dysphagia by identifying patients at risk for swallowing problems (see Fig. 1). Being identified as *at risk* of dysphagia following screening indicates the need for further assessment. A great variety of different types of screening has been described in the literature. Many screening tools consist of trial swallows using water in various aliquots or a range of different viscosities. Other forms of screening utilize combined protocols of trial swallows and pulse oximetry, pulse oximetry alone, evaluating clinical features (e.g. abnormal gag, voice alteration, or volitional cough), cervical auscultation, and elements of medical history (e.g. recurrent episodes of pneumonia or cough elicitation tests) [11, 12]. In addition, screening tools may use different endpoints to define being *at risk*, such as penetration, aspiration, pharyngeal delay, or dysphagia.

Screening tools using multiple swallow trials may differ in the order of selected viscosities or selected volumes per swallow trial. For example, the Toronto Bedside Swallowing Screening Test or TOR-BSST [13] consists of two steps; the first step is screening for abnormalities in voice quality and tongue movement, and the second step consists of ten consecutive teaspoons of water. Any failed item during either the first or second step constitutes a positive screen result and indicates an increased risk for dysphagia and a need to referral for assessment. Authors of water swallow screening tools may argue that the rationale for screening is solely to identify persons *at risk* of dysphagia and, therefore, no other consistencies need to be included[14].

Conversely, the volume–viscosity swallowing test or V-VST [15], another commonly used screening, uses three different viscosities (referred to as nectar, liquid, and pudding) and three volumes (5, 10 and 20 ml), resulting in a maximum of nine swallow trials. Whereas the TOR-BSST screens for being at risk for dysphagia only, the V-VST provides additional oral intake advice. Still, as only single trials are used per volume–viscosity combination, any advice on oral intake should be made with caution, as using a limited number of swallow trials can underestimate the aspiration risk in persons with dysphagia, especially when using boluses with a thin liquid consistency [16]. Even when a screening tool uses several trials of the same volume and viscosity, for example the Gugging Swallowing Screen or GUSS [17], any advice on oral intake should nevertheless be supported by findings from further assessment.

There is still no consensus on the order in which different viscosities should be offered when expanding the water protocol with, for example, thickened liquids, puddings, or solid boluses. Some authors start with thickened liquids using increased patient safety in terms of reduced frequency of aspiration as rationale, whereas other authors prefer starting with thin liquids referring to the heightened risk of residue when using higher viscosities [18]. Also, the use of different volumes may impact screening results; consecutive sips with large volumes in patients without overt airway responses or voice changes seem to rule out risk of aspiration, whereas small volumes with single sips indicate aspiration when clinical signs are present [14].

All patients at risk of dysphagia should be screened. Common populations at risk are frail older persons, stroke patients, patients with progressive neurological diseases (e.g. Parkinson’s disease or dementia), children and adults with non-progressive neurodevelopmental conditions (e.g. cerebral palsy), and patients with head and neck cancer [7, 19, 20]. Patients failing screening should be referred for further assessment by a dysphagia expert. However, patients having an established risk for dysphagia or manifest signs of dysphagia through case history may be referred for further assessment without being subjected to screening. If patients cannot be screened due to their cognitive state or health condition, screening should be postponed, usually resulting in recommended nil per os while waiting for screening. Some patient groups, however, may follow different screening and assessment pathways as no improvement in their condition is to be expected, for example, patients who may be at risk of dysphagia and have progressive cognitive decline (e.g. dementia). The lack of consensus and guidelines on how to manage these vulnerable populations result in moral dilemmas that need to be resolved on a case-by-case basis between healthcare practitioners and patients’ family. In comparison, screening is usually recommended to be performed within the first 24 h following the stroke [21]; however, there is no international consensus on what should be the critical timeframe for screening in other diagnostic groups.

### Selection Criteria for Screening

Screening needs to meet the following feasibility criteria before being implemented in clinical settings: simple administration, use of non-invasive methods to minimize distress to patients, and straightforward to train clinicians involved [22, 23]. While most screening tools are freely available, some, like the TOR-BSST, can only be used after attending an online training module to improve standardization and consistency in rating.

Screening tools need to be valid, reliable, and show good diagnostic performance. Screening tools can be developed and validated for specific target populations (e.g. patients after stroke, patients with head and neck cancer or frail older persons). The use of certain screening tools may not be recommended in other patient groups than the populations for which the screen was originally developed and validated for; *validity* of a screening must be confirmed again when its use is extended to other clinical populations. Sufficient intra-rater, inter-rater, or test–retest *reliability* is often expressed by an intra-class correlation coefficient (ICC) or weighted Kappa equal to or greater than 0.70 [24]. *Diagnostic performance* of a screening tool is determined by comparison with a reference test, usually fiberoptic endoscopic evaluation of swallowing (FEES) or videofluoroscopic evaluation of swallowing (VFS). According to the international COnsensus-based Standards for the selection of health Measurement INstruments (COSMIN), this is also referred to as criterion validity [25]. The reference test is considered the ‘gold standard’ and hypothesized to have no false negative or false positive results. In other words, the ‘gold standard’ correctly indicates the presence of dysphagia when the person truly has dysphagia (true positives) and identifies subjects as not having dysphagia when they truly do not have dysphagia (true negatives). As such, it is not recommended to use another screening or clinical assessment as reference test unless considered a ‘gold standard’. Furthermore, both reference tests and screening tools may use different endpoints. For example, when administering a FEES, the occurrence of aspiration may be used as endpoint to confirm the diagnosis of dysphagia, whereas a water swallow screening may use coughing or a wet voice as endpoint for identifying a patient at risk of dysphagia. As different endpoints yield different diagnostic performance, clinicians and researchers must predefine and select endpoints before determining diagnostic performance.

Diagnostic performance comprises elements such as sensitivity, specificity, positive and negative predictive value, positive and negative likelihood ratio, and diagnostic odds ratio (Table 1) [26]. As applied to the topic at hand, sensitivity and specificity refer to how well the screening tool identifies the presence and absence of dysphagia, respectively, whereas predictive values are the probability that a positive or negative test result is accurate. As such, sensitivity and specificity provide clinicians information about which screening to select, whereas predictive values inform the persons being tested about the chances of actually having dysphagia.

**Table 1:** Diagnostic performance: terms, acronyms, and definitions [26]

Term	*[Acronym]*	Definition	Formula^a^
Prevalence	*[PR]*	Proportion of subjects in a population having a disease	Diseased / Total population
Sensitivity	*[Se]*	Proportion of reference test positive (diseased) subjects who test positive with the screening tool	Se = TP / (TP + FN)
Specificity	*[Sp]*	Proportion of reference test negative (healthy) subjects who test negative with the screening tool	Sp = TN / (TN + FP)
Positive Predictive Value	*[PPV]*	Proportion of positive results in statistics and diagnostic tests that are true positive results	PPV = TP / (TP + FP)
Negative Predictive Value	*[NPV]*	Proportion of negative results in statistics and diagnostic tests that are true negative results	NPV = TN / (TN + FN)
Positive Likelihood Ratio	*[LR* +*]*	Probability of a person who has the disease testing positive divided by the probability of a person who does not have the disease testing positive	LR + = Se / (100 – Sp)
Negative Likelihood Ratio	*[LR-]*	Probability of a person who has the disease testing negative divided by the probability of a person who does not have the disease testing negative	LR- = (100 – Se) / Sp
Diagnostic Odds Ratio	*[DOR]*	Odds of a positive test in those with disease relative to the odds of a positive test in those without disease	DOR = LR + / LR-
Error		% of observations that were misclassified by the model	(FP + FN) / (TP + TN + FP + FN)
Accuracy		% of observations that were correctly classified by the model	(TP + TN) / (TP + TN + FP + FN) = 100 – error
Precision		Proportion of TP to all positive predictions	TP / (TP + FP) = PPV

^a^TP = True Positive = Identified patient; FP = False Positive = Healthy person identified as patient (Not identified healthy person); TN = True Negative = Identified healthy person; FN = False Negative = Patient identified as healthy person (Not identified patient)

Depending on work environment and requirements, different cut-offs for diagnostic performance may be used. For example, if assessment after screening is expensive or invasive, clinicians may prefer to minimize the number of false positives and use a cut-point with high specificity. Conversely, if the penalty for missing a case is high (e.g. the disease is fatal but can be treated successfully), a clinician may prefer to maximizing the number of true positives by using a cut-point with high sensitivity. In general, tests with high sensitivity have low specificity; they are good for identifying actual cases of the disease but often at the expense of high rates of false positives. For example, results from a meta-analysis showed that increasing volumes in water swallow tests resulted in higher sensitivity but lower specificity when screening for aspiration in stroke patients [27]. There are, however, no strict guidelines on the optimal cut-offs for diagnostic performance. Some diagnostic reviews only included screening tools with a high sensitivity (at least 70%) and at least a moderate specificity (at least 60%; e.g. [11, 12]). Even so, guidelines on what cut-off scores to use remain ambiguous.

A receiver operating characteristics (ROC) curve, a probability curve, visualizes the trade-off between sensitivity and specificity by presenting the true positive rate (sensitivity) as function of the false positive rate (100 minus specificity). Conversely, the area under the curve (AUC) can be interpreted as the average value of sensitivity for all possible values of specificity or the average value of specificity for all possible values of sensitivity [28]. AUC represents a measure of separability; how well is the model capable of distinguishing between two classes or groups, for example, between persons with or without dysphagia? The COSMIN guidelines stipulate that an AUC of at least 0.70 (or 70%) is required to provide evidence for sufficient criterion validity [24].

## Non-Instrumental Assessment

### Assessment in a Nutshell

After a patient has been screened for dysphagia and identified as being at risk for dysphagia, further assessment is required (Fig. 1). Both ‘gold standard’ assessments VFS and FEES can diagnose aspiration (including silent aspiration) and other physiological problems in the pharyngeal phase. However, access to instrumental assessment may be restricted and no international consensus exists regarding which visuoperceptual or software-based measures to use for analysis of these video recordings. Moreover, there is insufficient psychometric evidence to recommend any individual measure as valid and reliable to interpret VFS and FEES recordings [29].

Another step after screening is *non-instrumental clinical assessment* by a dysphagia expert. In the process of clinical assessment of dysphagia, many different aspects may be distinguished, for example, medical history: conducting a physical examination; the subjective description of the swallowing problem or patients’ complaints [30]; and the expert’s clinical observations during the interview and examination process. Clinical assessment may have the following purposes, single or in combination: to identify possible causes of the swallowing problems; to estimate the safety of swallowing and risk of aspiration; to support decisions on oral or alternative feeding routes; to identify the need for further assessment (e.g. instrumental assessment); and to establish baseline data for future comparisons after intervention or during the course of a disease [22]. Clinical assessment may also address patient needs that are specific to certain clinical populations. For example, in patients with head and neck cancer, the effects of radiation and chemotherapy may be important and need to be described in detail.

Clinical assessment may refer to a large variety of assessments, each of which may be describing different aspects of dysphagia. In the absence of systematic reviews, textbooks and current opinion papers provide different overviews. While there is currently no comprehensive overview of clinical assessments, most literature seems to agree on the following four categories: 1) assessment of cognition and communication; 2) evaluation of the oral, laryngeal, and pharyngeal anatomy, physiology, and function (including cranial nerve examination); 3) oral intake and nutritional status; mealtime observations; and 4) intervention trials (e.g. bolus modification, postural adjustments and/or swallow manoeuvers) [22, 31].

Another form of non-instrumental clinical assessment involves patient self-report measures as part of the multidimensional assessment of dysphagia. Patient self-report measures target two distinct but related concepts: *functional health status* (FHS) versus *health-related quality of life* (HR-QoL). FHS refers to the influence of a given disease (e.g. dysphagia) on particular functional aspects, whereas HR-QoL is the unique personal perception of someone’s health, taking into account social, functional, and psychological issues [32].

### Patient-Reported Measures

*Patient-reported measures* play an important role in patient-centred healthcare and can improve communication, patient engagement, and self-efficacy as patients may be more involved in goal setting [33–35]. The use of a patient-reported measure, however, may be limited by patients who experience cognitive and/or language comprehension deficits. Recently, a framework (patient-reported outcome measures or PROM-cycle) was developed to support the selection and implementation of patient-reported measures in individual patient care and for quality improvement [35]. The PROM-cycle describes eight steps necessary for systematic selection and implementation of PROMs in a cyclic approach and provides links to, for example, relevant tools and existing guidelines, examples from clinical practice and methodological considerations.

Patient self-report measures in dysphagia often combine both FHS and HR-QoL in the same measure, even though both are considered two distinct concepts. Consequently, disease-related functioning cannot be distinguished from disease-related quality of life as subjectively experienced by patients. Further, psychometric reviews on patient self-evaluation (e.g. [36, 37]) and psychometric evaluations on most commonly used self-report measures in dysphagia (e.g. [38–42]) identified that all existing measures have either poor psychometric properties or lack comprehensive evaluation and reporting of psychometric properties. This flags an urgent need for research. For example, even though the Eating Assessment Tool (EAT-10) is one of the most commonly used FHS measure in dysphagia, recent evidence using contemporary *Item Response Theory*^1^ (IRT; Rasch analyses [43, 44]) did not support its use in either research or clinical practice [38, 40–42]. Another frequently used FHS questionnaire is the Sydney Swallow Questionnaire (SSQ [45]), which is yet to be evaluated using IRT.

Among those self-report measures mainly targeting HR-QoL are the Swallowing Quality of Life questionnaire (SWAL-QOL [46]), the Dysphagia Handicap Index (DHI [47]), the Deglutition Handicap Index (DHI [48]), and the MD Anderson Dysphagia Inventory (MDADI [49]). The SWAL-QoL, like the EAT-10, is one of few measures that has been evaluated using Rasch analyses. As an outcome of the evaluation, it was recommended to further investigate the SWAL-QoL’s underlying structure and psychometric characteristics prior to continued clinical use [39, 50]. For most self-report measures, however, even though their use is common practice, their psychometric robustness is yet to be established using IRT [36, 37].

The use of self-report measures for *oral health* has gained importance in recent years. Poor oral health and concomitant dysphagia are important risk factors of aspiration pneumonia [51]. Oropharyngeal colonization by respiratory pathogens has shown to play a key role in the pathophysiology of aspiration pneumonia [52–56]. A recent systematic review reporting on patient-reported measures for oral health in adults identified 20 English-language multiple-item questionnaires summarized into four domains: oral function, orofacial pain, orofacial appearance, and psychosocial impact [57]. Examples of frequently used self-report measures in oral health include the Oral Health Impact Profile (OHIP [58]) and the Oral Health Questionnaire for Adults [59]. Most self-reported measures in oral health, however, need further psychometric evaluation before supporting their implementation in healthcare and research [57].

Given that *gastroesophageal reflux disease* (GERD) is common in populations presenting with dysphagia [2, 60, 61], self-report on GERD may form part of dysphagia assessment. Many examples can be found in the review by Bolier, Kessing [62] including, for example, the widely used and validated GERD Impact Scale (GIS [63]) and the Reflux Disease Questionnaire (RDQ [64]). Still, in the absence of further psychometric evaluations, many of these measures may not meet psychometric quality criteria [65].

### Assessment of Anatomy and Physiology

Before assessing the anatomy and physiology of the swallowing act, some higher cortical functions that act as precursors to oral feeding need to be evaluated. These include, but are not limited to, the patient’s alertness, responsiveness, cognition and language skills, as well as head and trunk control (i.e. motor control) [66]. In the absence of standardized formal assessments, *input–output reasoning* will inform further clinical assessment of functional anatomy (Table 2–3) whereby innervation (input) from a muscle or functional groups of muscles (effectors) will, in turn, result in deglutition-related actions (output).

**Table 2:** Simplified overview of effectors of swallowing: input–output reasoning for clinical assessment of functional anatomy [111]

Input ^a^*Afferents*	Effector	Output^a^ *Efferents*
V2 (maxillary nerve), V3 (lingual nerve: branch of inferior alveolar nerve of the mandibular nerve)	Lips	VII (Labial sphincter)
V3 (lingual nerve: branch of mandibular nerve)	Tongue	XII (Oral control of bolus)
V3 (mandibular nerve)	Jaw	V motor (Mastication)
V, IX	Soft palate	V, X (Palate function)
V	Mouth and cheeks	V motor, VII (Oral control of bolus)
IX	Base of tongue	XII (Propulsion into oropharynx)
X	Epiglottis (lingual side)	X (Laryngeal sphincter function)
X (superior laryngeal nerve)	Epiglottis (laryngeal side)	X (Laryngeal sphincter function)
X (superior laryngeal nerve)	Glottis and supraglottal larynx	X (Laryngeal sphincter function)
X (inferior laryngeal nerve)	Subglottal larynx	X (Laryngeal sphincter function)
X	Cervical trachea	X (Cough reflex)
V, IX, X	Naso-oropharynx	IX, X (Velopharyngeal sphincter function)
X	Hypopharynx	X (Propulsion, pharyngeal squeeze)

^a^Cranial Nerves: *I* Olfactory nerve, *II* Optic nerve, *III* Oculomotor nerve, *IV* Trochlear nerve, *V* Trigeminal nerve, *VI* Abducens nerve, *VII* Facial nerve, *VIII* Vestibulocochlear nerve, *IX* Glossopharyngeal nerve, *X* Vagus nerve, *XI* Accessory nerve, *XII* Hypoglossal nerve

**Table 3:** Simplified overview of input, effector, output, and clinical assessment for each functional group involved in deglutition (e.g. [109–111])

Functional group	Motor Input	Effector	Output
	*Innervation*^*a*^	*(Main) Muscles*	*(Main) Actions in Deglutition*
Masticatory	CN V	Lateral / Lateral pterygoid Masseter Temporalis	Mastication Closure oral cavity Mandible: raise
Facial	CN VII	Buccinator Orbicularis oris	Lip/mouth: seal Bolus: push towards teeth
Intrinsic tongue	CN XII	Inferior / Superior longitudinal Transverse Verticalis	Tongue: shorten – lengthen, narrow – broaden, tip up –down, concave – convex bow tongue Bolus: preparation, formation, positioning, transport
Extrinsic tongue	CN X	Palatoglossus	Tongue: protrude – retract, lower – raise Bolus: preparation, formation, positioning, transport Seal oral cavity
	CN XII	Genioglossus Hyoglossus Styloglossus	
Suprahyoid	CN V	Anterior belly of digastric Mylohyoid	Hyoid: lower – raise, protract – retract, stabilize Mouth floor: stabilize, elongate Mandible: lower
	CN VII	Posterior belly of digastric Stylohyoid	
	C1 [via CN XII]	Geniohyoid	
Infrahyoid	C1 [via CN XII]	Thyrohyoid	Hyoid: lower, stabilize Mouth floor: stabilize, elongate Larynx: raise – lower, stabilize
	C1-C3 [via Ansa cervicalis CN XII]	Omohyoid Sternohyoid Sternothyroid	
Palatal	CN V	Tensor veli palatine	Soft palate: raise – retract, lower, brace, tense Oropharynx entrance: widen Posterior tongue: raise Uvula: raise Seal back of oral cavity from oropharynx Seal nasopharynx
	CN X	Levator veli palatin Palatoglossus	
	CN XI [via CN X]	Uvular	
Pharyngeal	CN IX	Stylopharyngeus	Palate: lower Pharynx: raise, shorten Larynx: raise Seal oral cavity Seal nasal cavity Narrow pharyngeal lumen Bolus: transport Oesophageal sphincter: most distal component (of pharyngo-oesophageal segment or PES)
	CN X	Palatopharyngeus Salpingopharyngeus Superior / Middle / Inferior pharyngeal constrictor	
Laryngeal	CN X	Aryepiglottic Lateral / posterior cricoarytenoid Oblique / Transverse arytenoid Thyroarytenoid Thyroepiglottic	Vocal folds: adduct – open Arythenoids cartilages: approximate to epiglottis Epiglottis: lower Aryepiglottic folds^b^

^a^Cranial Nerves: *I* Olfactory nerve, *II* Optic nerve, *III* Oculomotor nerve, *IV* Trochlear nerve, *V* Trigeminal nerve, *VI* Abducens nerve, *VII* Facial nerve, *VIII* Vestibulocochlear nerve, *IX* Glossopharyngeal nerve, *X* Vagus nerve, *XI* Accessory nerve, *XII* Hypoglossal nerve

^b^Laryngeal sphincter function or squeeze: true vocal folds, false vocal folds, aryepiglottic folds

A strict order of events provides clinical markers that guide clinicians during assessment with a functional–anatomic pathway of motor outputs activated by sensory inputs (Table 2) [67]. Therefore, the anatomy and physiology that underpins the swallowing act need to be understood to allow for the assessment thereof. Swallowing is a highly integrated neuro-sensorimotor function, realized by organs (effectors) affiliated with different systems and inter-linked with other functions, so that swallowing, breathing, and phonoarticulation alternate according to hierarchically correlated pathways [68]. Swallowing is the result of external (sight and smell) and internal sensory stimuli (touch, temperature, and taste), which converge at the meduallary swallowing centre, from where the motor efferents of the bulbar *cranial nerves (CNs)* start: CN V (Trigeminal nerve: motor), CN VII (Facial nerve), CN IX (Glossopharyngeal nerve), CN X (Vagus nerve), CN XII (Hypoglossal nerve), and cervical spinal nerves C1-C3. The CN XI (accessory nerve) carries motor fibres for the sternocleidomastoid muscle and trapezius muscles (spinal root), and parasympathetic preganglionic visceral fibres (cranial root) that join the CN X reaching the soft palate, pharynx, larynx, and oesophagus. The brainstem swallowing centre is widely connected and receives cortical, subcortical, thalamic, limbic and cerebellar descending modulatory afferents, which transform the semi-reflex nature of swallowing into spatiotemporal integrated muscular contractions (muscular patterns) [5]. The neurological structures (including cortex, extrapyramidal and pyramidal system, and cranial nerves) are contained in the neurocranium (protective shell surrounding the brain and brain stem), while the swallowing effectors are contained in the viscerocranium (skeleton supporting facial structure). At this level, the upper parts of the respiratory and digestive tract are arranged in parallel and conjugate in the pharyngeal cavity, which becomes a common pathway for both air and bolus transport. In this way, the pharynx acts alternately as an organ of the respiratory tract (chamber for air passage) as well as an organ of the digestive tract (peristaltic contracting chamber for bolus passage).

Anatomy of the upper digestive tract allows the organization of functional chambers operating in a concatenated series [69]. In the oral cavity, the bolus is prepared (minced and mixed with saliva) as a consequence of coordinated actions between facial muscles, cheeks, teeth, jaws, tongue, and oral floor muscles [70]. When the bolus is transported into the pharyngeal cavity, a reflex reconfiguration of the pharynx occurs; the oropharynx transforms from a respiratory pathway to a swallow pathway [71]. The pharynx acts as a digestive lumen, generating contractile and peristaltic activities with the aim of transporting the bolus to the oesophagus [72]. A strict hierarchy between vital primordial reflex activities determines the arrest of respiration (apnea), thus allowing the pharyngeal transportation of the bolus [73]. The reconfiguration of the pharynx is defined by glossopalatal opening, velopharyngeal closure, upper and anterior hyoid laryngeal excursion, and upper oesophageal sphincter opening.

Articulation and voice quality are checked during speech and sustained vowel production and provide information about vocal fold or glottis closure. Furthermore, facial appearance and expression may indicate concerns about orofacial musculature, presence of surgical scarring, asymmetry, and inadequate labial seal (drooling). Touch, temperature, and taste sensibility can be tested on the lips, mucosa of the oral cavity, tongue (body, base, margins), fauci, soft palate, and posterior wall of the oropharynx using low-tech devices such as a wisp of cotton or tongue blade. The same low-tech devices can be used to test thermal and gustatory sensibility by wetting them in hot or iced water, or in salty, sweet, bitter, sour, or sparkling solutions.

Tone and strength of the tongue can be assessed by eliciting resistance against a tongue blade, while mobility is assessed by administering non-articulatory or articulatory praxis tasks. The opening of the jaws (temporomandibular joint) is closely examined and masticatory muscles are evaluated for its praxis and strength. The pterygoid muscles are tested during lateral mandibular movements with the jaw applying resistance against a hand, while the masseter and temporal muscles can be palpated during contraction. The symmetrical soft palate movement is evaluated using a simple articulatory task (i.e. by sustaining the vowel /a:/) or during the process of testing sensibility. The gag reflex can be elicited by stimulating the back of pharynx with a tongue blade/probe. Finally, the presence of the pharyngeal reflex is elicited by asking patients to perform a dry swallow while the clinician holds three fingers on the external hypo-laryngeal axis; the index finger is placed on the hyoid bone, the middle finger is placed on the thyroid notch, and the ring finger is placed on the cricoid ring. There should be an antero-superior displacement of these structures of about 2 to 2.5 cm during a dry swallow. Table 3 provides additional examples of clinical assessment of functional anatomy and physiology of the swallowing act.

### Clinical Swallowing Evaluation

A *clinical swallowing evaluation* (CSE) is typically performed after screening for swallowing problems to provide additional information to ascertain the relative risk for aspiration and to direct clinical decision making [74]. CSE findings are important in determining whether an instrumental assessment may be required (if not already performed) and to decide which compensatory strategies and postural manoeuvres need to be prioritized for trialling to inform dysphagia therapy planning and patient management. At least a tentative diagnosis must be made and a management plan agreed upon following a CSE [31]. However, aspiration and other physiological problems in the pharyngeal phase of swallowing can only be diagnosed through directly observation using instrumental assessments [75–77].

The initial step in a CSE is to compile a thorough case history with the patient and/or carer. This involves a careful review of the medical history, if available, a history of the present condition (e.g. onset, duration of difficulties, symptoms), current swallowing status (e.g. method of oral intake and current diet, strategies that help swallowing, easiest and most difficult food consistencies to swallow), and factors that might influence management (e.g. comorbidities, cognition, food restrictions and nutritional status, presence of gastroesophageal reflux, and cultural preferences) [30]. Common examples of the many available measures used while compiling a case history are the Functional Oral Intake Scale (FOIS; [78]) and the Mini-Mental State Examination (MMSE; [79]) or Mini-Cog [80] to screen for cognitive impairment.

After compiling the case history, the clinician forms a preliminary clinical hypothesis that is tested during ensuing assessment. Only a few standardized CSE tests are available across dysphagia populations. The Mann Assessment of Swallowing Ability (MASA [81]) is an example of a standardized CSE. The MASA was validated on stroke patients and has adaptations for patients with head and neck cancer (MASA -C [82]). While conducting a CSE, the clinician also completes a clinical observation of the patient noting body posture, head posture at rest, level of alertness, and ability to follow assessment instructions, upper respiratory tract secretions, and the patient’s ability to manage his/her saliva. In addition, an orofacial examination is conducted that includes a cranial nerve examination. Oral health may be assessed using a measure such as the Oral Health Assessment Tool (OHAT [83]).

Trial feeding is only considered an appropriate management strategy if the patient has an adequate voluntary cough and is able to manage his/her own secretions. If, however, the patient is drowsy, medically unstable, and unable to swallow saliva requiring suctioning, then trial feeding is deemed too high risk and should not be attempted. Self-feeding is preferred over feeding by carers or health care providers, to mimic typical eating and drinking patterns. As mentioned earlier, there are opposing views regarding which consistency to trial first. Some clinicians prefer to begin trials with small 5 ml volumes of water, gradually increasing the volume before moving to thicker consistencies. The rationale is that water, if aspirated, combined with good oral hygiene will be absorbed into the lungs without the immediate risk for the development of pneumonia [84, 85]. Other clinicians prefer to start with thickened consistencies so as to reduce the risk for aspiration [15]. Trials with fluids that promote sensation, for example carbonation and sweet and sour bolus, may be included in CSE [86–88]. The temperature of the bolus may also be manipulated with colder boluses used to increase sensation [89]. Thicker consistencies may be trialled later as they may give rise to residue in the pharynx which, in turn, can lead to aspiration [18]. When assessing chewing it is important to evaluate the patient’s ability to manage solids and to identify risk of choking also. Assessments such as the Test of Mastication and Swallowing of Solids (TOMASS [90]) may be useful.

When possible, a CSE should also include mealtime observation. This is particularly useful in patients with cognitive impairments where feeding and swallowing difficulties may be influenced by fluctuating levels of attention, fatigue, or environmental factors (e.g. background noise or other distractors). Observations such as the patient’s ability to self-feed, the need to use adaptive eating utensils, duration of the meal, and the presence of fatigue are noted. Examples of available standardized CSEs based on mealtime observation are the McGill Ingestive Skills Assessment (MISA [91]) for older persons with feeding difficulties, and the Dysphagia Disorder Survey (DDS; [92]) developed and validated for persons with developmental disabilities. Both measures have demonstrated promising, robust psychometric properties [91, 93, 94].

During the CSE, individualized trials are conducted to investigate the effectiveness of compensatory strategies the person may already have put in place or to test new strategies and examine which are most effective for safe and efficient oral intake. Compensatory strategies comprise changing *what* the person eats (i.e. diet modification) and changing *how* the person eats (e.g. head posture, alternate liquid/solid swallows, volume control). The efficacy of implementing adaptive equipment (e.g. beakers, modified spoons, straws) in promoting safe oral intake should also be examined.

Ancillary to conducting CSE is evaluation such as pulse oximetry and cervical auscultation. Pulse oximetry measures peripheral capillary oxyhemoglobin saturation and is used to detect a decrease in saturation, which is indicative of aspiration during swallowing [95]. Cervical auscultation is hypothesized to differentiate between normal and abnormal swallowing by listening to the sound of swallowing and swallowing-related respiration using a stethoscope placed on the neck [96]. However, there is contention regarding diagnostic accuracy of pulse oximetry in predicting aspiration and current evidence does not support its use [95]. Cervical auscultation, although commonly used, is equally contentious and there is limited evidence to support its use [96].

Limitations of the CSE are that protocols vary and that there is variability in practice even within the same clinical settings and between populations [97–99]. However, its key strength is providing low-cost clinical information to the multidisciplinary team in the immediate management of patients with dysphagia, especially if clinical teams do not have access to instrumental assessments.

### Challenges and Minimum Requirements for Assessment

A *core outcome set* (COS) is an agreed minimum set of outcomes that should be measured and reported in clinical trials of a specific disease or target population [10]. When selecting outcome measures as part of a COS, four criteria need to be considered [10]. First, both the construct to be measured (e.g. FHS, HR-QoL) as well as the target population (e.g. age, diagnosis) must be clearly defined. Second, existing measures must be identified by, for example, performing literature searches or using recent systematic reviews. Third, the quality of all measures must be evaluated both in terms of quality of psychometric properties as well as feasibility for administering the measure within particular setting. Fourth, one measure must be selected for each outcome or construct in a COS (Fig. 1).

The COSMIN group established an international consensus-based taxonomy, terminology, and definitions of *measurement properties* for health-related patient-reported outcome measures [25]: guidelines for using the taxonomy, including a standardized appraisal tool for rating methodological quality of psychometric studies [100], *criteria for evaluating psychometric quality* of assessments [101, 102], and a rating system for synthesizing and grading psychometric evidence [101, 102]. The taxonomy distinguishes nine psychometric properties across three domains (Table 4): validity (content validity, structural validity, cross-cultural validity, hypothesis testing for construct validity and criterion validity), reliability (internal consistency, reliability and measurement error), and responsiveness. When selecting an assessment, all nine psychometric properties are considered when identifying the assessments with the most robust psychometric properties.

**Table 4:** Psychometric domains and properties according to the COSMIN taxonomy

Domain	Measurement property	Definition^a^
*Validity*		***Degree to which an instrument measures the construct(s) it purports to measure***
	*Content validity*	Degree to which the content of an instrument is an adequate reflection of the construct to be measured
	*Structural validity*	Degree to which the scores of an instrument are an adequate reflection of the dimensionality of the construct to be measured
	*Hypotheses testing for construct validity*	Degree to which the scores of an instrument are consistent with hypotheses based on the assumption that an instrument validly measures the construct to be measured
	*Cross-cultural validity*	Degree to which the performance of the items on a translated or culturally adapted instrument are an adequate reflection of the performance of the items of the original version of the instrument
	*Criterion validity*	Degree to which the scores of an instrument are an adequate reflection of a ‘gold standard’
*Reliability*		***Degree to which the measurement is free from measurement error***
	*Internal consistency*	Degree of the inter-relatedness among the items
	*Reliability* (intra-rater, inter-rater, test–retest)	Proportion of the total variance in the measurements which is because of ‘true’ differences among patients
	*Measurement error* (intra-rater, inter-rater, test–retest)	Systematic and random error of a patient’s score that is not attributed to true changes in the construct to be measured
*Responsiveness*		***Ability of an instrument to detect change over time in the construct to be measured***
Interpretability^b^		Degree to which one can assign qualitative meaning to an instrument’s quantitative scores or change in scores

^a^Definitions derived from Mokkink, Prinsen [24]

^b^Interpretability is considered an important characteristic of a measurement instrument but is not a psychometric property

Assessments that have poor psychometric properties should not be used in either clinical settings or research. Minimum requirements for including an outcome measure in a COS are high-quality evidence for good content validity and good internal consistency [10]. However, as recent psychometric reviews have identified missing or indeterminate evidence for many psychometric properties of existing assessments in dysphagia (e.g. [29, 36, 37]), often only preliminary conclusions on the psychometric robustness of the assessments can be drawn based on current available psychometric evidence in the literature.

Content validity, the degree to which the content of an assessment is an adequate reflection of the construct to be measured, is considered the most important psychometric property within the COSMIN taxonomy [102]. Concerns around content validity are raised when assessments that have been developed and validated for specific target populations are used in different populations. For example, the MD Anderson Dysphagia Inventory (MDADI) is a dysphagia-specific quality of life questionnaire targeting patients with head and neck cancer [49], whereas the Dysphagia in MUltiple Sclerosis questionnaire (DYMUS) is a FHS self-report assessment for adults with multiple sclerosis [103]. Assessments like the MDADI and DYMUS that have been developed and validated for a specific clinical population cannot be used in another population without the risk of compromising its content validity [102]. For an assessment to have good content validity [102], all items need to be aligned with the construct of interest in a specific population and context of use (relevance), all key concepts of the construct need to be covered (comprehensiveness), and instructions, items and response options need to be understandable to the target population as intended (comprehensibility).

Problems may also occur when using a translated version of an original assessment, without undertaking the required steps to ensure good cultural validity. The proposed framework of translation by Koller, Kantzer [104] is based on a review of influential translation guidelines and summarizes the reconciliation processes that needs to be undertaken when translating measures. The framework stipulates that through a process of reconciliation, two or more independent forward translations are merged into one single translation using decision criteria on comprehensibility, cultural appropriateness, grammar, and use of relevant and consistent terminology. Next, cross-cultural validity needs to be determined by comparing the performance of items from a translated or culturally adapted assessment with the original assessment version [24].

Finally, aspects of *feasibility* need to be taken into consideration when deciding on which assessments to select for measuring outcomes. Feasibility refers to the practicability of using an assessment in its intended setting, taking into account constraints of time, money, and interpretability [10]. Other important factors related to feasibility include, for example, patient’s cognitive functioning and physical ability, patient’s and clinician’s ability to comprehend items, ease of administration, duration of administering the assessment, simplicity of score calculation, accessibility of required equipment in different settings, copyright requirements, and associated costs to acquire and administer the assessments [10]. Another consideration include administering, where appropriate, assessments via telehealth instead of face-to-face to increase accessibility to healthcare in rural and remote areas [105], to increase service reach, reduce costs [106], and more recently to continue providing safe health services during the COVID-19 pandemic.

## Recommendations and Future Research

### Selection of Screening and Assessments

Where access to instrumental assessment is restricted, the use of clinical assessments becomes exponentially important when evaluating patients’ illness trajectories. There are many screening and non-instrumental assessments available for evaluating dysphagia in adults (see Table 5). However, even though measures may be part of daily clinical practice, their use may not be warranted due to poor diagnostic performance or lacking robust psychometric properties.

**Table 5:** Examples of commonly used screens and assessments for dysphagia in adults

Domain	Screening and assessment^a^	Acronym	Respondent	Reference
Standardized screening				
At risk of swallowing problems	Gugging Swallowing Screen Toronto Bedside Swallowing Screening Test Volume-viscosity swallowing test	GUSS TOR-BSST V-VST	Clinician Clinician Clinician	Trapl et al., 2007 Martino et al., 2009 Clavé et al., 2008
Standardized assessments				
Cognition & Communication	Mini-Cog	–	Clinician	Borson, 2000
	Mini-Mental State Examination	MMSE	Clinician	Folstein et al., 1975
Oral intake status	Functional Oral Intake Scale	FOIS	Clinician	Crary et al., 2005
Dysphagia-related quality of life & Functional health status	Dysphagia Handicap Index MD Anderson Dysphagia Inventory Sydney Swallow Questionnaire Swallowing Quality of Life questionnaire	DHI MDADI SSQ SWAL-QOL	Patient Patient Patient Patient	Silbergleit et al., 2012 Chen et al., 2001 Dwivedi et al., 2010 McHorney et al., 2002
Oral health status	Oral Health Assessment Tool Oral Health Impact Profile Oral Health Questionnaire for Adults	OHAT OHIP –	Clinician Patient Patient	Chalmers et al., 2005 Slade et al., 1994 WHO, 2013
Gastroesophageal reflux disease	GERD Impact Scale Reflux Disease Questionnaire	GIS RDQ	Patient Patient	Jones et al., 2007 Shaw et al., 2001
Swallowing	Mann Assessment of Swallowing Ability	MASA	Clinician	Mann et al., 2002
Mastication	Test of Mastication and Swallowing of Solids	TOMASS	Clinician	Huckabee et al., 2018
Mealtime observation	McGill Ingestive Skills Assessment Dysphagia Disorder Survey	MISA DDS	Clinician Clinician	Lambert et al., 2003 Sheppard et al., 2014
Non-standardized assessments				
Anatomy / Cranial nerve integrity	Clinical examination of the tongue, hard and soft palate, teeth, gums, oral mucosa, trigeminal (V), facial (VII), glossopharyngeal (IX), vagal (X), and hypoglossal (XII) cranial nerves.			
Oral motor skills / Physiology	Clinical examination of oral muscle strength, range, tone, steadiness, accuracy and coordination. Mealtime observation including observation of drooling or sialorrhea, mastication, eating speed, cough or choking, oral residue, head and body positioning.			
Trial feeding Intervention trials / Compensatory strategies	Bolus modification, postural adjustments and/or swallow manoeuvers			

^a^No international consensus exists on which screen or assessment for dysphagia in adults is preferred. In addition, many screens and assessments have unknown or poor psychometric properties. The presented list of screens and assessments does not provide a comprehensive overview, but examples of common clinical practice.

Therefore, based on this ESSD white paper, the following *recommendations* are made (Fig. 1):

1. Discontinue the use of non-validated dysphagia screening tools. Instead, use screening tools with good diagnostic performance, good reliability and validity and tools that meet feasibility criteria.
2. Implement screening using tools that have optimal diagnostic performance in selected populations that are at risk of dysphagia. These populations include, but are not limited to, stroke patients, frail older persons, patients with progressive neurological diseases (e.g. Parkinson’s disease or dementia), persons with cerebral palsy, and patients with head and neck cancer.
3. Discontinue the use of measures with insufficient or poor psychometric properties. Instead, use measures that demonstrate robust psychometric properties that meet psychometric quality and feasibility criteria.
4. Provide quality training in dysphagia screening and assessment to all clinicians involved in the care and management of persons with dysphagia.

### Future Research and Clinical Practice

There are great challenges for *future research* in the screening and assessment of dysphagia in adults:

- Existing measures in dysphagia with incomplete or missing evaluations of psychometric properties (i.e. validity, reliability, and responsiveness), need urgent and ongoing study.
- New measures need to be developed by using contemporary psychometric standards and methods, such as item response theory in combination to classic test theory.
- To ensure adequate content validity, studies should be conducted at the onset of developing a new measure to reach consensus on underlying definitions of constructs and to ensure that the items to be include are relevant and comprehensible and the measure is comprehensive in capturing the underlying construct(s).
- There is a need for robust studies that examine the costs and benefits of screening and assessment by telehealth versus face-to-face administration.
- There is an urgent need to reach *international consensus* on several crucial topics. Delphi studies^2^ would be an appropriate method to aim for consensus among experts and stakeholders on the following topics [107, 108]:
- A universally accepted definition dysphagia?
- How to define and measure severity of dysphagia?
- What is the minimum set of outcomes (COS) that should be measured and reported in dysphagia?
- Which outcome measures should be selected for each outcome in the minimum set of outcomes (COS)?
- What are the critical time points for administering dysphagia screening and assessment?

Instrument development and international consensus will enhance research designs and outcome measurement in future clinical trials and subsequently lead to improved health care pathways for patients with dysphagia. There is a need for standardized screening and assessment, and consistency in terminology and definitions used in research and clinical practice. When addressing the validity, reliability, and responsiveness of outcome measures, both measurement properties and criteria for good psychometrics should be defined (e.g. the COSMIN framework). Finally, when referring to dysphagia, a common language is needed to define its symptoms and promote consistent use of terminology in screening, assessment and interventions.

## Footnotes

1 Methodologies and interpretation of Classic Test Theory (CTT) findings are easier to interpret than those of Item Response Theory (IRT); however, the CTT framework has some limitations. In IRT the unit of analysis and results are not restricted to the test population, whereas in CTT the evaluation of psychometric properties is specific to the test population. In addition, IRT evaluates the reliability of each individual item, while CTT assesses the performance of a measure as a whole [43]. IRT models estimate both item and person parameters within the same model, calculate person-free parameter estimation and item-free trait level estimation, and identify optimal scaling of individual differences based on the evaluation of differential item functioning [44].

2 The Delphi technique is a structured process which aims to develop group consensus on a defined topic through a series of survey rounds [107]. The same participants complete each round (although some individuals may discontinue participating). Rounds are held until consensus is reached (or it becomes apparent that consensus cannot be reached). Results from previous rounds are used to inform participants about changes that may facilitate consensus in subsequent rounds [108]. Using the Delphi technique enables large number of individuals across diverse locations and areas of expertise to be included anonymously, thus avoiding domination of the consensus process by one or a few experts [107].
